# The role of auditory nerve innervation and dendritic filtering in shaping onset responses in the ventral cochlear nucleus

**DOI:** 10.1016/j.brainres.2008.09.054

**Published:** 2009-01-09

**Authors:** Christian J. Sumner, Ray Meddis, Ian M. Winter

**Affiliations:** a MRC Institute of Hearing Research, University Park, University of Nottingham, NG7 2RD, UK; b Centre for the Neural Basis of Hearing at Essex, Department of Psychology, University of Essex, Colchester, CO4 3SQ, UK; c Centre for the Neural Basis of Hearing, The Physiological Laboratory, Downing Street, Cambridge, CB2 3EG, England, UK

**Keywords:** Onset, Stellate, Cochlear nucleus, Point-neuron, PSTH, Rate-level functions

## Abstract

Neurons in the ventral cochlear nucleus (VCN) that respond primarily at the onset of a pure tone stimulus show diversity in terms of peri-stimulus-time-histograms (PSTHs), rate-level functions, frequency tuning, and also their responses to broad band noise. A number of different mechanisms have been proposed as contributing to the onset characteristic: e.g. coincidence, depolarisation block, and low-threshold potassium currents. We show that a simple point neuron receiving convergent inputs from high-spontaneous rate auditory nerve (AN) fibers, with no special currents and no peri-stimulatory shifts in firing threshold, is sufficient to produce much of the diversity seen experimentally. Three sub-classes of onset PSTHs: onset-ideal (OI), onset-chopper (OC) and onset-locker (OL) are reproduced by variations in innervation patterns and dendritic filtering. The factors shaping responses were explored by systematically varying key parameters. An OI response in this model requires a narrow range of AN input best frequencies (BF) which only produce supra-threshold depolarizations during the stimulus onset. For OC and OL responses, receptive fields were wider. Considerable low pass filtering of AN inputs away from BF results in an OL, whilst relatively unfiltered inputs produce an OC response. Rate-level functions in response to pure tones can be sloping, or plateau. These can be also reproduced in the model by the manipulation of the AN innervation. The model supports the coincidence detection hypothesis, and suggests that differences in excitatory innervation and dendritic filtering constant are important factors to consider when accounting for the variation in response characteristics seen in VCN onset units.

## 1 Introduction

Cells have been found in the ventral cochlear nucleus (VCN) that respond primarily to the onset of a stimulus (Pfeiffer, 1966; Godfrey et al., 1975, Bourk 1976) with a precisely timed first spike (Rhode and Smith 1986). However, the exact character of the onset response shows great variety. These cells also respond to a wider range of frequencies than AN fibers, show firing rate changes across a much wider range of sound levels, and often respond more strongly to broad band noise than pure tones (Rhode and Smith 1986; Winter and Palmer, 1995; Jiang et al., 1996; Palmer et al., 1996). They are predominantly innervated by the auditory nerve which is very well understood, yet they respond very differently to sound. These differences and the variety of their responses makes them very interesting examples of signal-processing by single neurons.

Onset cells have been divided into three sub-types, based on their PSTH responses to pure tones at best frequency (best frequency pure tone; BFPT): i. onset-ideal (OI) which respond only at the stimulus onset; ii. onset-later activity (OL; L has also variously been associated with ‘late’, ‘low’, or simply the shape of the response PSTH) which respond strongly at the onset with a single spike, and show some sustained firing during the rest of the stimulus; and iii. onset-chopper (OC), which respond with 2-4 regular chops at the stimulus onset (Rhode and Smith 1986). However, within the PSTH sub-types, there is great diversity (Rhode and Smith 1986, Winter and Palmer 1995).

The OC response has been associated with stellate cells (Smith and Rhode 1989), which project through the dorsal cochlear nucleus (DCN) and out along the intermediate acoustic stria (IAS; Smith and Rhode 1989, Smith et al., 2005, Oertel et al., 1990; Ferragamo et al., 1998). Because of their dorsally projecting axons Oertel et al. (1990) have termed these cells D-stellates. The dendrites of the cells are arranged in a plane perpendicular to the projection of AN fibers through the VCN, with fibers contacting in large numbers on the soma and on the distal dendrites (Smith and Rhode 1989). Thus, they receive innervation from a wide range of AN BFs (best frequencies). The membrane properties of OC cells are not well known. Stellates in general show a linear relationship between voltage depolarization and injected current (Wu and Oertel 1984; Oertel 1983). However, there is evidence that OC and chopper cells differ in their intrinsic currents (Oertel et al., 1990).

OI and OL responses have been associated with octopus cells (Godfrey et al., 1975; Rhode et al., 1983; Rouiller and Ruygo 1984; Feng et al., 1994), which occupy a restricted area of the posterior VCN (Osen 1969; Brawer et al., 1974; Hackney et al., 1990). Like D-stellate cells, they have thick dendrites lying across the field of AN fibers, and receive inputs from many AN fibers (Liberman 1993). In octopus cells, intrinsic currents are known to be essential for onset responses. OL and OI responses have also been associated with bushy cells (Rouiller and Ruygo 1984, Smith and Rhode 1987; Smith et al., 1991). These cells have densely branching dendrites, but most of the AN synapses appear to be onto the cell bodies (up to fifty). Bushy cells are also known to have specialized membrane currents (Oertel 1983, Manis 1990) and produce primary-like (PL), primary-like with-notch (PL-N) and occasionally even chopper responses (Rouiller and Ruygo 1984), suggesting that intrinsic currents, innervation and morphology affect responses in a complex way.

Although anatomical evidence suggests that the OC responses are from D-stellates and the OL responses are from bushy cells and octopus cells, evidence from single electrode recordings are not so clear. All types of onset have on average much wider receptive fields and wide dynamic ranges than AN fibers (Winter and Palmer 1995), and can show considerable temporal integration (10 ms-20 ms) of inputs (Palmer and Winter 1996). These properties are hard to reconcile with the observation that innervation of bushy cells is mostly onto the cell body. Presumably this would limit the levels of dendritic filtering and the range of AN BFs possible for innervation. Long integration times are also incompatible with our knowledge of octopus cells (Golding et al., 1995). The key to understanding these discrepancies may lie in an understanding of the underlying mechanisms. Several mechanisms have been proposed to explain onset responses. The most simple of these is coincidence detection: convergence of a large number of AN fibers produces a reliable input which is only supra-threshold at the stimulus onset, due to the adapting characteristic of the AN. This is supported by the extensive AN innervation found on D-stellate and octopus cells. Intrinsic currents can also contribute to onset responses. Such currents are well established in octopus cells (Golding et al., 1999; Bal and Oertel, 2000, 2001).

A number of modeling studies have looked at the effectiveness of different mechanisms for the onset response, and the sources of differences between different PSTH types. Arle and Kim (1991) used a MacGregor (1987) computational model to reproduce a number of basic VCN responses. For an onset response a membrane-voltage dependent threshold shift prevented sustained activity. Meyer (1993) investigated coincidence detection, inactivation and inhibition as mechanisms for onset production. In his simulations, coincidence detection was sufficient for OC responses, but OL responses required inactivation. More recently, Kipke and Levy (1997; Levy and Kipke 1997, 1998) showed that a threshold shifting mechanism inherent in the channel kinetics and coincidence detection could produce the onset response. They also found that if a smaller number of input fibers produced the same total current, the response tended to shift from an OC to an OL. Kalluri and Delgutte (2003a,b) have investigated models of onset cells in terms of their PSTH BF tone responses and rate-level (RL) functions. Like Kipke and Levy, they noted that a decrease in the number of input fibers would shift a model's response from OC to OL. The temporal precision of resulting OL models was, however, worse than the OC models. They proposed that if entrainment (the ability of a cell to respond on every cycle of a click train or low frequency pure tone) were taken as a constraint for OL or OI cells, then an additional stimulus dependant refractoriness was required. Kalluri and Delgutte (2003a) also looked at the effect of receptive field size on RL functions for pure-tones and broad band noise. They found that the rate of growth of the response for pure tones decreased with the width of the receptive field, but not for broad band noise.

Cai et al. (1997, 2000) have investigated models of the intrinsic currents found in octopus cells and demonstrated their importance producing onset responses. Rothman and Young (1996) investigated the processing of bushy cells in the VCN. These cells show mainly AN like activity, but employ inactivation currents in a manner similar to octopus cells. Some cells also show enhanced synchronization over AN fibers. They showed how this arose from the convergence of several AN fibers. They also were able to reproduce OL responses with this model given enough fibers.

Previous modeling studies have resorted at some point to an intrinsic current or inhibitory mechanism that assists the onset response. However, none have been convincing in the necessity of intrinsic currents, as they did not explore any other possibilities. We were interested in the possibility that dendritic filtering might also contribute to the character of the onset response. The model we have implemented here is a leaky integrate and fire device, which receives inputs from exclusively high spontaneous rate (HSR) auditory nerve fibers. Its simplicity allows for a comprehensive exploration of the functional effects of innervation position and density. Here we show that the model reproduces the different sub-classes of onset units despite the absence of deliberately applied threshold shifts, inactivating currents or inhibition. Importantly, we show that a difference in dendritic low-pass filtering, which can arise though differences in morphological properties of the dendrite and in particular the location of innervation along the dendrite (Rall, 1977), can determine the sub-class. We also show how the variety of BF pure-tone RL functions can be reproduced by controlling the number and BFs of the HSR AN fibers inputting to the cell. AN innervation patterns which do not fall off monotonically in fiber density away from unit BF, can account for a wider range of rate responses than previously modeled. We note additionally however, in agreement with Kalluri (2001) that these changes cannot effect the variation in observed rate responses to broad band noise.

**Fig. 1:**
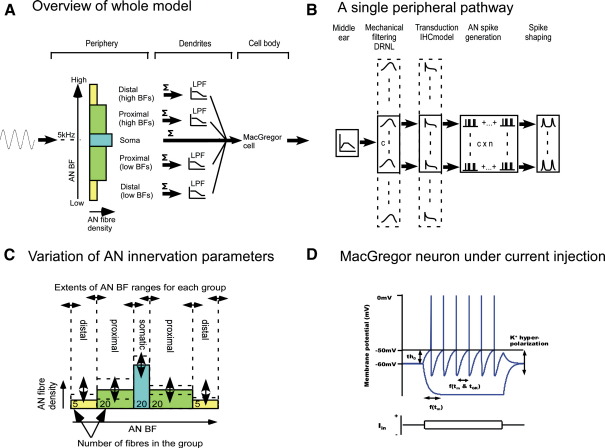
The architecture of the model. (A) Overview of the model shows 3 stages: peripheral inputs in five groups, dendritic filtering and summation, followed by a MacGregor integrate and fire model of spiking. Five groups of input fibers originate from contiguous channels of the AN model, each with different BFs. Each group innervates a different location on the cell dendrites. (B) A single group within the peripheral stage consisting of a bandpass middle ear function, several channels of the basilar membrane model at different contiguous points, and an IHC for each channel. For each channel *n* auditory nerve fiber inputs are generated depending on the model parameters, and the resulting impulses filtered to resemble post-synaptic potentials. (C) Schematic of the parametric variation of AN inputs. Each group of inputs is varied independently. The parameters are the BF range of each group and the density of fibers. (D) Example voltage trace from the MacGregor neuron under current injection at depolarizing (upper voltage trace showing spike generation) and hyperpolarizing (lower voltage trace) current values. The time course of current injection is shown separately below the voltage traces.

Fig. 1A shows a schematic of the entire model. From a model of the auditory periphery, a population of AN fiber outputs is generated. The AN fibers are grouped into five pathways on the basis of their BFs. The AN projection through the VCN is tonotopically arranged. The dendrites are assumed to be arranged across the path of the fibers such that AN BFs vary monotonically with the point of innervation on the cell. Thus these pathways represent both the somatic AN inputs and the inputs to the proximal and distal dendrites from AN fibers above and below the cell's BF. The inputs to the cell are low-pass filtered, independently for each pathway, to mimic the effects of dendritic filtering for innervation at different locations on the cell. The result is then summed and is the input to a basic model of somatic leaky integration and spike generation. The output is the resulting stream of detected spike events. Details of the implementation are given in section 5.

## 2 Model results

### 2.1 Histograms of pure tone responses

#### 2.1.1 Classic response types

**Fig. 2:**
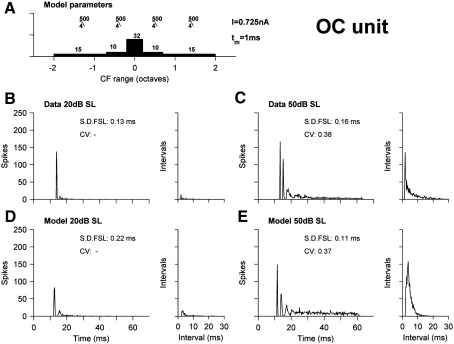
Onset-chopper BFPT PSTH and ISIH responses. (A) Schematic of model parameters. Histogram shows density of distal, proximal and somatic pathways. *I* is the current for each fiber, τ*_m_* is the membrane time constant. The fraction above each pathway indicates the cut-off and order of the dendritic filters. (B, C) An OC neuron. PSTH and ISIH responses are shown at 20 dB SL (B) and 50 dB SL (C). (D, E) Corresponding model responses. All histograms are generated using 250 stimulus presentations, and bin-widths of 0.2 ms. There is no LPF icon for the ‘somatic’ pathway as there is no dendritic low-pass filtering.

The first test of the model is its ability to reproduce the three basic sub-classes of onset response. Fig. 2, panels B and C, show a classic onset-chopper (OC), recorded using the methods described in section 5.4. At low stimulus levels (here 20 dB above threshold; Fig. 2B), the PSTH response is a single clear spike at stimulus onset. For higher intensities (Fig. 2C), a second onset spike, or chop, emerges, followed by some sustained activity. The interspike-interval histograms (ISIHs) for this cell are also shown. The ISIH at 20 dB SL (sensation level; Fig. 2B) has a single small peak, reflecting that the many presentations elicit at most one spike and only occasionally two. At 50 dB SL (panel C) this cell has a sharp peak at the interval associated with the initial chop, decaying to longer intervals for the sustained activity. Panels D and E show the response of a model, where the parameters of the model have been adjusted to reproduce the data. The model is close to the data in most respects, although the chopping interval is visibly wider than the data. Fig. 2A shows the parameters that led to this behaviour. There are 82 AN fiber inputs, from a 4-octave range of BFs, and these inputs are low pass filtered (order 4, cut-off 500 Hz). The response of this model is easily understood in terms of the strength of input at a given time. At low levels, only the AN fibers near to BF are firing, and because the inputs are adapting only the initial portion of their response is supra-threshold. As the level grows, the response at stimulus onset grows enough to drive the cell to threshold twice before the inputs adapt. Sustained activity also becomes supra-threshold. Fig. 2 also shows the coefficient of variation (CV; when there are enough spikes) and standard deviation of first spike latency (S.D. FSL) for both cell and model. The discrepancy for the FSL S.D. is larger at 20 dB SL than it is at 50 dB SL.

**Fig. 3:**
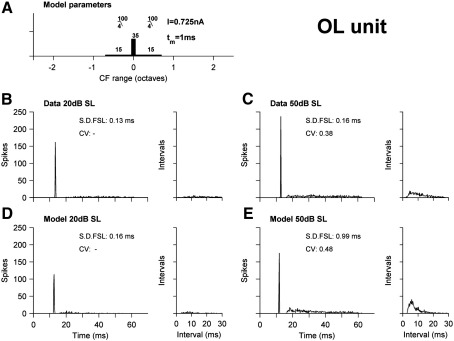
Onset-locker BFPT PSTH and ISIH responses, as per Fig. 2. (A) Schematic of model parameters. (B, C) An example OL unit. (D, E) Model responses for the parameters shown in panel A.

Fig. 3 shows the responses from a typical onset-later activity (OL) unit and a model closely mimicking its responses. Panels B and C show the BFPT response PSTHs and ISIHs at 20 dB and 50 dB SL respectively. OLs differ from OC units, in having only one onset spike for high intensity stimuli (compare Fig. 3C with 2C). It also lacks the corresponding peak in the ISIH (Fig. 3C). Figs. 3D–E show responses of the model fitted to this cell, which again bears close resemblance to the data. Panel A is a schematic of the parameters. Unlike the OC model, the AN inputs with BFs different to that of the cell have been heavily low-pass filtered (order 4, cut-off 100). Inputs grow with level, but because they are smoothed and delayed, they do not contribute to the onset and do not produce any chopping. In Fig. 3 the correspondence of CV and S.D. FSL between cell and model is quite good, except at 50 dB SL the onset in the model is considerably more variable than the data.

**Fig. 4:**
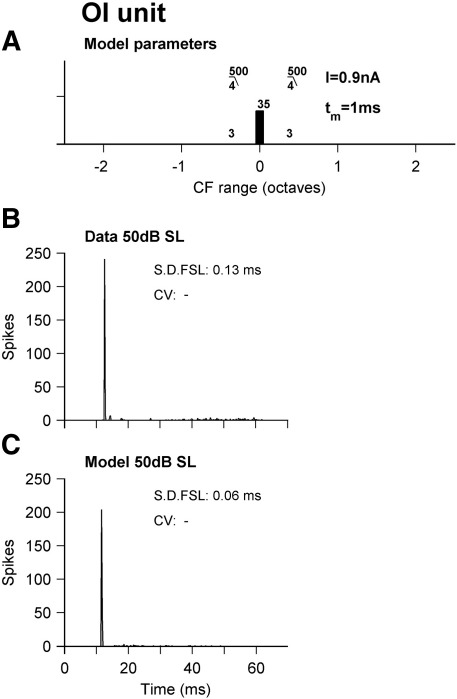
Onset-ideal BFPT PSTH and ISIH responses. (A) Schematic of model parameters. (B) An example OI unit at 50 dB SL. (C) Model response at 50 dB SL for the parameters shown in panel A.

The onset-ideal (OI) class gives a pure onset response even at the highest sound levels tested. Fig. 4 shows an example of an OI unit (Fig. 4B) and a model (Fig. 4C) which reproduces this behavior. Panel A shows the model that reproduces this response. To reproduce this response AN fiber inputs must come from a narrow range of BFs, and the cell must only reach firing threshold near to the level of saturation for the AN. This narrow BF range is at odds with the known anatomy of some of the cells associated with OI responses, and will be discussed below.

**Fig. 5:**
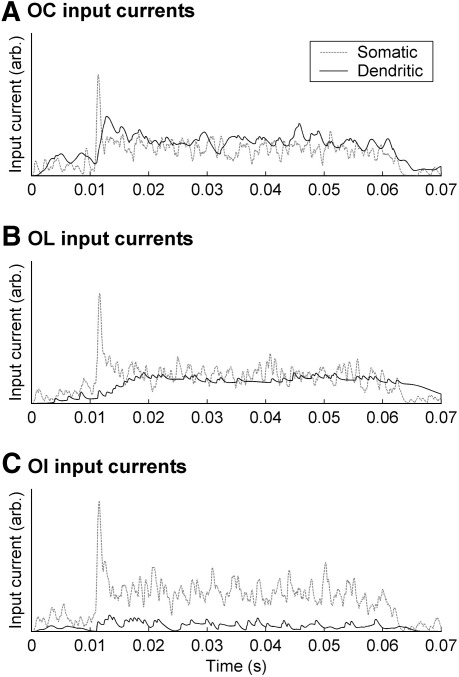
The somatic and dendritic inputs (in arbitrary units of current) to the MacGregor integrate and fire stage, for each of the models shown in Figs. 2–4. Dashed lines show the inputs applied directly to the soma and the solid lines show the inputs from the dendrites (the sum of the filtered proximal and distal inputs). All responses are for a BF tone input at 50 dB SL. (A) Inputs to the OC model in Fig. 2; (B) OL model shown in Fig. 3; (C) OI model shown in Fig. 4.

Some insight into how the basic models produce their responses can be gleaned from considering the sub-threshold processing of the inputs for these three examples. Fig. 5 shows separately the somatic and dendritic inputs to the MacGregor stage of the models, for a 50 dB SL BFPT. The somatic, on-BF, inputs are simply the sum of all the auditory nerve fiber inputs (only filtered to mimic the time course of short post-synaptic potentials), whilst the off-BF dendritic inputs have been subject to additional low-pass filtering. The proximal and distal inputs have been summed together. In the case of the OC example (panel A), both somatic and dendritic inputs are similarly strong. The dendritic inputs are only slightly smoothed and delayed. In the case of the OL example (panel B) the dendritic inputs are heavily filtered and slowly grow in strength. Thus they cannot contribute to the onset response. In the OI example, even though the level is high, the dendritic inputs are very weak.

### 2.2 Rate-level functions

**Fig. 6:**
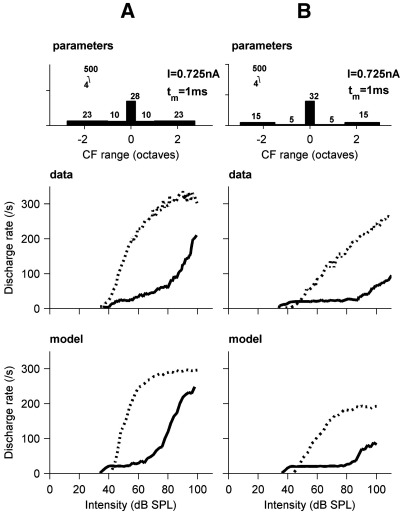
Rate-intensity functions for BFPT (solid lines) and broad band noise (dotted lines). (A and B) are different example of RL functions from onset units (middle row) and corresponding model responses (bottom row). The top row shows schematics of the parameters for the two models. Filter characteristics apply to all dendritic pathways (somatic pathway is not filtered).

The diversity of onset units extends to rate-level (RL) functions, both for BFPT stimuli, and for broad band noise (BBN). Fig. 6 shows example RL functions in two cells (middle panels in A and B) for BFPTs (solid lines) and for BBN (dashed lines) from Winter and Palmer (1995). Pure tone responses are sloping, and do not show saturation even at the highest levels. Some show reasonably constant growth, but responses can also ‘plateau’: firing rates remain at one (20 spikes/s for a 50 ms tone) spike per-stimulus for a 40 or 50 dB range of sound levels, before then rising steeply. The response to broad band noise is even more diverse. For some cells (not shown) the rate follows that of the pure-tone response. In other cases, the response to broad band noise can be quite different in slope (Fig 6, middle panels). The rate for BBN often exceeds the pure tone response, whilst the thresholds for both are usually similar. BBN responses in the AN contrast with this: they have higher thresholds, and generally elicit less response.

The lower panels in Fig. 6 show the rate responses of models. The pure-tone responses (solid lines) are very reproducible, by manipulation of the receptive field in the same way as the PSTH responses. The parameters used for the two units are shown in the top panels. The parameters were tuned manually to resemble the data as closely as possible. The distance of an AN input BF from the unit BF determines the level at which it starts to contribute to the cell response.

The BBN responses of the model (dotted lines) are favourable simulations of the response to broad band noise. They differ from their AN inputs, in that the thresholds are often close to that of BF pure-tone responses. Also the rate of growth of activity can be fast and the highest firing rates are very high. However, the model responses to BBN all share the same steep and saturating RL function as a consequence of the simple nature of the integration of inputs in the model. As a result the model cannot produce gently saturating RL functions for BBN shown in A, the straight BBN RL functions as shown in B, or plateauing BBN RL functions (Winter and Palmer, 1995; not shown).

## 3 Systematic variation of model parameters

In section 2 it was shown that a simple model with no intrinsic ion currents beyond a spiking mechanism can show many of the response types observed in onset cells in the VCN. The only manipulations made were to the arrangement of AN inputs and the degree of dendritic filtering. In this section we explore how these manipulations led to the different responses by systematic variation of the parameters.

**Fig. 7:**
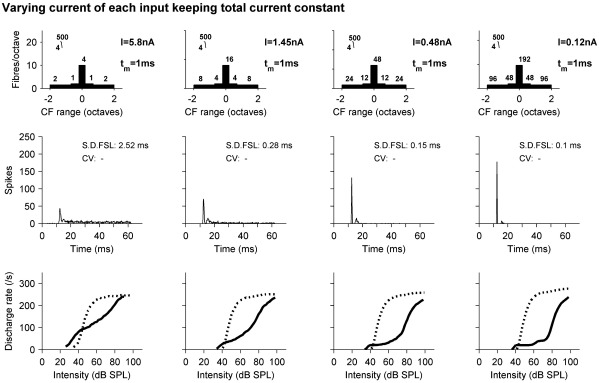
The effect of a varying the number of AN input fibers whilst keeping the total amount of current constant. Each column shows the responses of a different model with the total number of input fibers increasing from left to right. Top row shows schematics of the models. Filter characteristics apply to all dendritic pathways (somatic pathway is not filtered). Middle row shows response PSTHs at 20 dB SL, and bottom row shows the RL functions for BFPTs (solid line) and BBN (dotted line).

Fig. 7 shows how the convergence of a large number of small inputs compares with a small number of large inputs, in terms of the pure tone response PSTHs at 20 dB SL (middle row) and rate level functions (RLFs) for both pure tones (lower row; solid lines) and BBN (lower row; dots). In each column a different model is shown, with the parameters displayed in the top row. The model in the left-most column has only 10 AN input fibers, whilst the rightmost column has a total of 480 input fibers. In all cases the total input current from all fibers is almost identical and no other parameters change. As the degree of convergence increases the steady state response is reduced and the onset response grows. This effect of the number of fibers is similar to that previously shown by Kalluri and Delgutte (2003a,b). Likewise the pure tone RLF drops for low level pure tones with the increasing number of fibers. This figure demonstrates the value of convergence of a relatively large number of small AN inputs in producing a robust onset-type response.

**Fig. 8:**
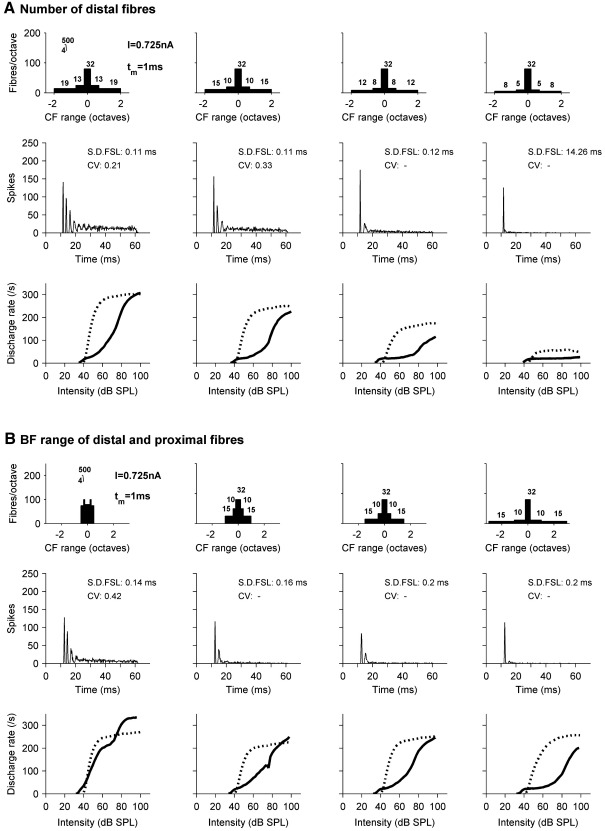
The effect of varying the range and number of off-BF fibers. (A) Shows how PSTHs at 20 dB SL and RL functions to BFPTs (solid line) and BBN (dotted line) vary with the number of off-BF fibers synapsing on the dendrites. The number of fibers decreases from left to right. The number of somatic input fibers and all other parameters remain constant. (B) Shows how the BF range of inputs affects the PSTH at 20 dB SL and RL functions. The BF range of dendritic inputs increases from left to right.

Fig. 8A shows the effect of varying the number of fibers on the proximal and distal dendrites, keeping all other parameters constant including the number of somatic inputs. PSTHs at 20 dB SL, and RL functions in response to pure tones (solid line) and BBN (dashed line) are affected. The leftmost column represents models with more dendritic fibers, and produces too many spikes at 20 dB SL to be classified as an onset unit according to the criteria of Winter and Palmer (1995). The model in the far right column has little dendritic innervation and the response to pure tones is of the OI type. Notice that whilst RL functions to tones and noise gradually reduce in their high level spike rate, the response to tones changes in character, with an extending plateau corresponding to 1 spike per stimulus, whereas for BBN stimuli the effect is a reduction in the saturated rate without any change in the overall shape and only a small rise in the threshold.

Fig. 8B shows the effect of varying the range of BFs covered by the AN inputs, without changing the number of fibers or total input current. The format is the same as that of Fig. 8A. A small BF range narrows the dynamic range, producing a steeper pure tone RL function and a PSTH at 20 dB SL that would not be classified as an onset type. A large range has little effect on the BBN RL functions, but the pure tone RL function develops a plateau covering a range of approximately 20 dB, and the maximum firing rate is slightly reduced.

Thus, Fig. 8 shows that in order for a model neuron to display onset properties, it must either have weak inputs from a narrow BF range, producing the OI type, or receive innervation from a wide BF range so that the pure tone response at low levels is an onset only. It also shows how these manipulations, when they do affect the responses to BBN, largely only affect the saturated rate.

**Fig. 9:**
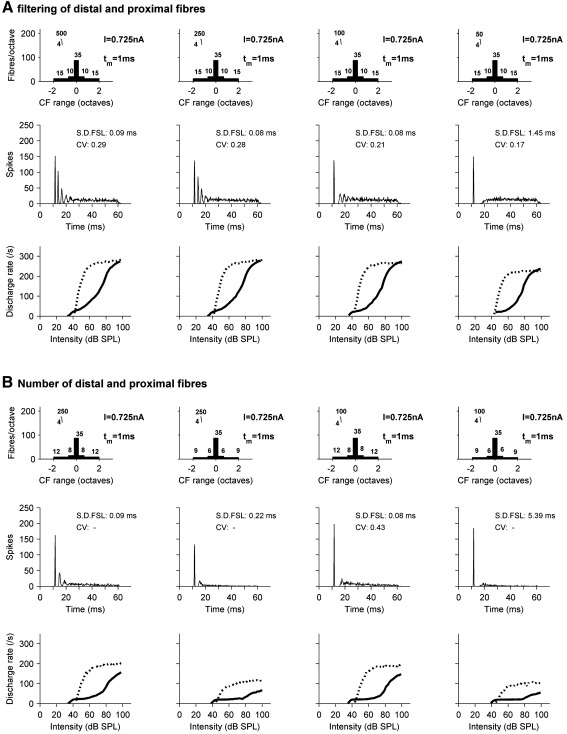
The role of low-pass filtering dendritic inputs in determining whether response sub-class is OC or OL, and the shapes of the RL functions. (A) Shows how PSTHs at 50 dB SL (second row) and RL functions (third row) to BFPTs (solid line) and BBN (dotted line) vary as the low-pass cut-off of the dendritic filtering is reduced from left (500 Hz) to right (50 Hz). All other parameters remain constant. (B) Left 2 columns show the effect of reducing the number of dendritic AN inputs from panel A, when the dendritic filter cut-off is 250 Hz. Right 2 columns show the effect when the dendritic filter cut-off is 100 Hz.

Fig. 9 explores how low pass filtering of dendritic inputs and the number of dendritic input fibers determine whether the response PSTH at 50 dB SL is OC or OL, and also shows the effect of RL functions. In Fig. 9A the cut-off frequency of the low pass dendritic filters is systematically reduced from 500 Hz as in the OC example (Fig. 2) to 50 Hz, which is lower than the 100 Hz used for the OL example (Fig. 3). More filtering smoothes and delays dendritic inputs, reducing the amount of integration during the onset of AN inputs. Thus the response shifts from OC to OL type. Although the PSTH shapes change dramatically, there is only a subtle shift in the RL functions. As more of the dendritic inputs are filtered out, there is a slight lowering in firing rate. Fig. 9B shows the effect of reducing the number of dendritic inputs once the low-pass cut-off has been reduced. The left two panels of Fig. 9B take the model shown in the 2nd column of Fig. 9A, which has a dendritic filter cut-off of 250 Hz, and reduces the number of dendritic inputs. Notice that in Fig. 9A, column 2, the model has strong chopping and is of OC type. Reducing the total number of dendritic inputs from 50 to 30 changes this response to OL. Similarly, the right hand columns of Fig. 9B reduce the number of dendritic fibers when the dendritic cut-off is 100 Hz. Both of these models show OL responses. Thus the OL response can arise through a combination of more dendritic filtering and less dendritic input. These factors are complementary to the total number of input fibers shown previously (Kalluri and Delgutte 2003a,b, Kipke and Levy 1997, and Fig. 7 here) to determine whether response types were OL or OC. And of course additional currents are also likely to play a role, especially in octopus and bushy cells. As in Fig. 9A, the filtering has very little effect on the RL functions for either tones or noise.

**Fig. 10:**
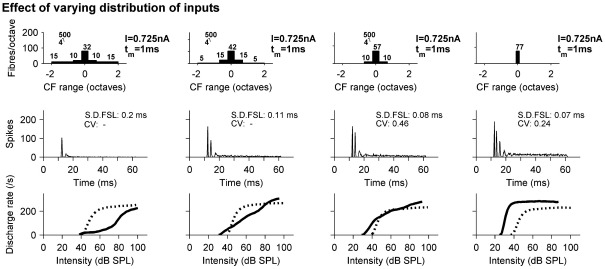
The role of BF range in determining PSTH type (for a BFPT at 20 dB SL; middle row) and RL functions (bottom row) and thresholds for BFPT (solid lines) and BBN (dashed lines). Each column shows the RL functions from a different model, with distribution of input fibers both in terms of location of innervation and BF decreasing from left to right.

Fig. 10 demonstrates how the spread of inputs across a wide range of BFs affects the relative thresholds for tones and noise, and also how the PSTHs for a BFPT at 20 dB SL are affected. A characteristic of onset units is that, unlike the auditory nerve, the thresholds are quite similar for both BF tones and BBN. This is because onset units integrate energy across a wider range of frequency than the auditory nerve. The model reproduces this result, as shown in Fig. 10. The leftmost panel is the OC example from Fig. 2 and in each subsequent model the inputs shift from the dendrites to the soma. Thus the balance of fibers and overall tuning shifts towards a narrow BF range. The RLFs for BBN hardly change at all. However, the response to BF tones changes from one with a very shallow slope at low levels to a steep, saturating function and a low threshold. This increase in firing rate at low-tone levels is also reflected in the PSTHs, which change from an onset response to a sustained chopper response (note the low CV).

## 4 Discussion

The model supports the adequacy of coincidence for onset responses, without the need for additional currents. This is particularly true of the OL class. It also reproduces the wide variety of RL functions for BF-tones and some for broad band noise. The models are also consistent with the data in that PSTH types (OL versus OC) and pure tone RL function types are not apparently related. By removing many of the complexities of other models we have been able to concentrate on functional significance of dendritic processing, and explore parameter space more thoroughly. This has revealed more clearly the scope and the limitations of dendritic processing for reproducing VCN onset responses.

The model, however, is unrealistic in some important aspects. It is effectively a model of stellate responses, as it lacks the specialized membrane properties of bushy and octopus cells. There are none of the non-linear effects associated with sub-threshold depolarisation in even a basic compartmental model, such as Kipke and Levy (1997). Intracellular measurements of OC cells show a sustained depolarisation which is insufficient for firing in chopper cells (Smith and Rhode 1989), suggesting some threshold shift may be occurring. There is also evidence of inhibition shaping responses (Palombi and Caspary 1992). The strength of the model is that, despite missing all these characteristics it still accounts for so much of the data. It suggests that AN innervation is sufficient for forming onset responses in the VCN and that differences in AN innervation can account for much of the variation.

The deficiencies of the model are also informative. Parameter exploration makes it clear where the omitted mechanisms might have a crucial role to play. The most dramatic failure of the model is for broad band noise (BBN) RL functions. In response to BBN all input fibers are stimulated equally, so the cell input resembles a very strong HSR fiber input. This cannot produce RL plateaus in a model that fires as a monotonic function of input. This might suggest a role for fibers of different thresholds, inhibition, some inactivation mechanism, or a combination of mechanisms. It might also suggest a role for inputs other than HSR auditory nerve fibers. The auditory nerve model used here also reproduces realistic RL functions for medium- and low-spontaneous rate auditory nerve fibers, which are quite different to HSR fibers. It was however beyond the scope of this study to explore this possibility.

Another consistent shortcoming is that the initial chopping interval of OC responses is too long. Lowering the integration time constant (τ_*m*_) reduces the interval, but it also increases the sustained discharge rate (not shown). A peri-stimulatory threshold shift of some kind would reduce this problem. Justification for this may lie in intracellular recordings. Under current injection, D-stellates frequently show a two-component recovery after an action potential (Oertel et al., 1990).

In this model, OI responses require a narrow receptive field or AN inputs will cause the inputs to grow. Experimentally, many OI units respond across wide frequency ranges (Smith and Rhode 1989). This suggests that a narrow band OI response is possible with a coincidence mechanism alone. Narrow tuning would be expected in bushy (Rouiller and Ruygo, 1984; Smith et al., 1991) cells. However, this limitation of the model suggests that wide-band OI responses, such as those originating from octopus cells, would require additional mechanisms.

Kipke and Levy (1997) and Kalluri and Delgutte (2003a) found that an OL model can be created from an OC by using a smaller number of fibers with stronger input strengths. This was also evident in our models. However, the precision of the onset must also be affected. Kalluri and Delgutte (2003a,b) have proposed that OI and OL responses required an additional stimulus dependant refractory time. This was to support entrainment, which requires very precise onsets. We did not test entrainment due to the lack of evidence at 5 kHz BF, to which the model was restricted. Strong entrainment has only been seen in low-BF neurons (Rhode and Smith 1986; Godfrey et al., 1975). In our model low-pass filtered inputs contribute to sustained activity but not the onsets. They can therefore produce a precise- single-onset spike and sustained activity. Furthermore, the main evidence for entrainment is from the posterior VCN, so these units may have been octopus cells. Kalluri and Delgutte's proposed mechanism might be functionally equivalent to the membrane currents found in octopus cells.

Our simulations suggest low-pass filtering could be a factor in determining whether a response would be OC or OL. In the model, the response in the first few milliseconds depends on the strength of the inputs, and the degree of low-pass filtering. If the input at onset is weak, the neuron will reach threshold only once before the AN adapts. Dendritic filtering confers a delay, and changes the shape of the depolarisation, which renders input ineffective during the onset of the tone. Thus OLs are more likely to be produced by a model with severe dendritic filtering. There is good theoretical evidence that dendritic transmission affects the shape of post-synaptic potentials (PSPs) (Rall 1977, Major et al., 1994). Palmer and Winter (1996) examined the temporal integration of two-tone inputs, with different frequencies and different onset times, in VCN onset cells. They found temporal integration windows were typically in the range of 10-20 ms. They did not report any differences between OC and OL units. However, there are many factors that affect this in the model. The number of fibers is one determinate, as discussed already. Also, OC response could not be produced if the membrane time constant (τ_*m*_) was 2 ms or more (not shown here). Further, the range of frequencies of off-BF tones used by Palmer and Winter (1996) was limited by the time for which a neuron could be held. One neuron (an OC) was held for five hours. It showed great variation in temporal integration at different frequencies. Given the difference in complexity between dendritic fields that can arise in nature and this simple model, predictions must be drawn very carefully.

This model cannot be taken as evidence against other mechanisms for onset production. Given that cytoarchitectonic details do shape the responses of cells in the way that this and other studies suggest, then the variation in the degree of branching, the extent and orientation of dendrites within a morphological class, suggest that many different patterns of innervation of a neuron should produce different responses. But it is also clear that membrane currents are often shaping responses. Our model is not meant as a model of the responses of bushy cells or octopus cells, and so comparison with these is not appropriate. Numerous previous models (Meyer, 1993; Kipke and Levy 1997, Levy and Kipke 1997, Kalluri (2001) have been successful in reproducing both OL and OC responses. All these models have convergence of large numbers of inputs and differ widely in their tuning, and this confers on them the basic characteristics of broad tuning and a phasic response. The models differ mainly in their mechanism for producing OL responses. Our models demonstrate that neither intrinsic currents nor the degree of coincidence are crucial in determining responses. They also show more realistic rate-level functions for pure tones, ISIHs, first spike precision and even two-tone facilitation (although not shown here). None of these have been modeled accurately in previous studies. However, no models have shown to be successful in reproducing well the responses to BBN. This is logically therefore the next challenge, and may offer insights into the real constraints of different mechanisms.

The success of this simple model raises interesting general questions about cells in VCN. One issue is: which features of a neuron actually contribute to the responses? The coincidence detection mechanism relies on the adaptation of the auditory nerve input, so in a sense the essential mechanism for producing an onset occurs at the neurons' inputs. However, without convergence of many inputs, stochasticity would obscure the onset. The broad BF range of inputs is not necessary for the onset response (as seen in Fig. 4), but produces pure tone RLFs with a wide dynamic range. Although we have not described the tuning properties of these models, most do show the broad tuning seen in onset cells. Thus the properties of the responses result from an interaction of cell processing and properties inherent in cells' inputs. A second issue is the extent to which cells in the VCN can be considered as coming from separate classes. There are some clear anatomical distinctions. For example, octopus cells occupy a restricted region of posterior-VCN and D-stellate cells project only within VCN and to the VCN on the opposite side. However, in terms of PSTH response type, RLFs and tuning, our models clearly vary along a continuum from onset to sustained choppers. This suggests that real cells in VCN might also be less distinct than is implied by classification schemes (Blackburn and Sachs 1989, Winter and Palmer 1995).

## 5 Experimental procedures

### 5.1 AN model

The AN is simulated with a model of the guinea-pig cochlea (Sumner et al., 2003b, Holmes et al., 2004). The model employs a dual resonance non-linear (DRNL) filterbank to simulate the non-linear mechanical filtering in the ear (Meddis et al., 2001). Neural transduction is performed with a model of the inner-hair-cell (Sumner et al., 2002, 2003a). We describe them only briefly here.

Fig. 1B shows the different stages in peripheral model for one of the five pathways. The stimulus waveform is input to a linear bandpass filter, reflecting the frequency response of the middle ear. The output from the middle-ear stage is fed into the DRNL filterbank. Each channel of the filterbank itself consists of two parallel band-pass branches whose outputs are summed. One is linear, whilst the other has a broken stick compression function, sandwiched in-between two cascaded stages of filtering. The response from this branch is linear at small input amplitudes, and becomes compressive at higher levels. This architecture can reproduce many of the characteristics of basilar membrane (BM) measurements (Meddis et al., 2001). Longitudinal variation in tuning and compression arises from variation in the parameters with BF. Filterbank BFs are spaced evenly in log-frequency across the entire filterbank.

The output from the DRNL filterbank is BM velocity, and each channel drives an inner hair-cell (IHC) stage. The IHC stage incorporates fluid-cilia coupling, a simple passive equivalent electrical circuit of the IHC receptor potential, and calcium controlled release of neurotransmitter into the cleft. The adaptation characteristics of the auditory nerve are modeled as pre-synaptic depletion of neurotransmitter available for release, as it cycles around three reservoirs. When employed with the DRNL filtering stage, this model can reproduce many of the effects observed on the AN and their variation with fiber type, including realistic RL functions at and away from BF, adaptation and phase-locking. Here it will provide only high-spontaneous rate (HSR) AN fiber inputs to the VCN model. The IHC model as previously described releases neurotransmitter vesicles stochastically. To speed simulations the model is run deterministically and output from the IHC stage is taken as transmitter release probability. From this, multiple fiber outputs are generated by an independent Poisson random number generator, which is modified with an absolute refractory period of 1 ms. The resulting spike events are 20 μs rectangular pulses. These are filtered, with linear 2nd order low-pass filters with a cut-off of 600 Hz. This shapes them to have a time course and duration (around 1 ms) similar to synaptic transmission. Current size is a parameter that can vary, but once set is equal for all inputs.

Fig. 1C shows schematically the arrangement of AN fibers for the five pathways. Each pathway is specified by the range of filterbank BFs, expressed relative to a nominal neuron BF of 5 kHz, and the total number of AN fibers in the pathway. The choice of a 5 kHz BF was a purely practical one, to limit the number of parameters. It also fixed the BF above the phase-locking limit. The IHC synapse outputs are divided exclusively among the five pathways, and the same number of fibers is generated for each filterbank channel within a single pathway. The entire filterbank has a constant density of channels in log frequency. Therefore, an algorithm is used to search for the combination of total filterbank density and number of fibers for each pathway. An error of 5% in the number of fibers for each path is considered acceptable. This produces an arrangement which is as computationally efficient as possible whilst not compromising behavior.

### 5.2 Cell model

The shaped spike trains of the auditory nerve model form the input to the cell model itself. These are summed into a single channel for each pathway, and low-pass filtered to simulate dendritic filtering. The filter order and cut-off frequency can vary for each channel. Normally, the ‘somatic’ inputs receive no filtering at this stage. The dendritic filtered inputs are summed and form the input current, *I*_*s*_, to the spiking generation part of the neuron (see Fig. 1A).

Spike generation is simulated using a MacGregor (1987, point neuron 10, p.458) point neuron model, similar to that used by Hewitt et al. (1992) to model VCN chopper cells. Essentially, this is a leaky integrate and fire model. The instantaneous membrane depolarization, *E*(*t*), above resting potential, *E*_*0*_, is described by the differential equation:

(1) $$\frac{dE\left(t\right)}{dt}=\frac{-E\left(t\right)+I_{s}\left(t\right)R_{i}+G_{k}\left(t\right)\left[E_{k}-E\left(t\right)\right]}{{\text{τ}}_{m}}$$

where *I*_*s*_(*t*)*R*_*i*_ is the depolarization due to an injected current to the soma *I*_*s*_(*t*) facing a constant input impedance of *R*_*i*_, τ_*m*_ is the membrane time constant, and *E*_*k*_ is the reversal potential of the potassium conductance *G*_*k*_(*t*). The potassium conductance is described by

(2) $$\frac{dG_{k}\left(t\right)}{dt}=\frac{-G_{k}\left(t\right)+bs}{{\text{τ}}_{Gk}}$$

where τ_*Gk*_ is the time constant of potassium conductance decay, *b* is the maximum potassium conductance, and *s* is the spiking variable (0 or 1). *s* is 1 if *E*(*t*) > *Th*_*0*_ and 0 otherwise. The output from the model is described by *p*(*t*) = *E*(*t*) + *s*[*E_b_* – *E*(*t*)]. Note that in this study, the threshold is a fixed value, *Th*_*0*_, which is not normal for this model. The parameters are given in Table 1. The values used are almost identical to Hewitt et al. (1992), except that the membrane time constant is lower (1 ms).

Fig. 1D shows example output of the neuron model, when stimulated with a constant positive and negative current injection. Under positive current injection, the membrane depolarizes. When the depolarization exceeds the fixed threshold, an action potential is generated. This initiates the ‘potassium current’, which hyperpolarizes the membrane potential to below resting. Under acoustic stimulation the input to the MacGregor stage is simply a current injection of the summed dendritic filter outputs from all five pathways. Thus, the spike generation stage is electrically de-coupled from the dendrites. Action potentials are detected with a thresholding algorithm to generate an output of spike events for analysis.

### 5.3 Simulation methods

All the model code has been implemented in C under the Development System for Auditory Modeling (DSAM) library, and the Auditory Model Simulator (AMS) application derived from it^1^. MATLAB was used as a harness for control of executables, manipulation of parameters and analysis of output.

The model is tested for its ability to reproduce the variety of different responses seen across the population of onset cells in the VCN. Three basic parameters are free to vary for each pathway: i. the BF range of the AN inputs, ii. the number of AN fibers, iii. the cut off of dendritic low-pass filtering. The peak current of input APs is also varied. The variation in these determines the behavior of the model. Simulations are conducted across a wide range of model parameters to test the model thoroughly. The models presented here represent a very small sample of the total number of simulations run during the development and testing of the model. All simulations were run with a time step of 10 μs. The experimental paradigm used to model BFPT responses closely follows that of Winter and Palmer (1995). Quoted values for regularity (CV) of models and data are the mean CVs from 25-33 ms after the start of the tone.

### 5.4 Physiological PSTHs

Unless otherwise stated the PSTHs presented in this paper were obtained routinely from studies in the guinea pig cochlear nucleus (e.g. Pressnitzer et al., 2001) in the Physiological Laboratory, Downing Street, Cambridge. The procedures used were approved by the United Kingdom Home Office (1986) Act by the issue of a project and a personal license to the third author. The details of the surgical preparation and stimulus presentation can be found elsewhere (e.g. Pressnitzer et al., 2001; Winter et al., 2001). In brief, when a single unit was isolated the BF and threshold at BF were determined using audio-visual criteria. The spontaneous discharge rate was measured over a 10 s period. The PSTHs were generated in response to 250 short tone-bursts (50 ms) at the unit's best frequency. Rise-fall time was 1 ms (Cos2 gate) and the repetition rate was 4/s. The starting phase of each tone burst was varied randomly to reduce the influence of phase-locked discharges on the shape of the PSTH for units with low best-frequencies.

## Figures and Tables

**Table 1:** MacGregor neuron parameters

*b*, potassium conductance (nS)	0.17
τ_*Gk*_, potassium time constant (s)	0.2e-3
*Th*_*o*_, Firing threshold (mV)	10
τ_*m*_, membrane time constant (s)	1e-3 (default)
*R*_*i*_, input resistance (Ω)	33e6
*E*_*b*_, cell reversal potential (mV)	0
*E*_*k*_, potassium reversal potential (mV)	− 10
*E*_*0*_, resting potential (mV)	− 60

## Footnotes

1 These can be downloaded from dsam.org.uk.
